# Neural Correlates of the Preserved Inhibition of Return in Schizophrenia

**DOI:** 10.1371/journal.pone.0119521

**Published:** 2015-04-13

**Authors:** Yingying Tang, Yan Li, Kaiming Zhuo, Yan Wang, Liwei Liao, Zhenhua Song, Hui Li, Xiaoduo Fan, Donald C. Goff, Jijun Wang, Yifeng Xu, Dengtang Liu

**Affiliations:** 1 First-episode Schizophrenia and Early Psychosis Program, Division of Psychotic Disorders, Shanghai Mental Health Center, Shanghai Jiao Tong University School of Medicine, Shanghai, China; 2 Shanghai Key Laboratory of Psychotic Disorders, Shanghai Mental Health Center, Shanghai Jiao Tong University School of Medicine, Shanghai, China; 3 Department of Psychology, East China Normal University, Shanghai, China; 4 Psychotic Disorders Program, UMass Memorial Medical Center, University of Massachusetts Medical School, Worcester, Massachusetts, United States of America; 5 Nathan Kline Institute for Psychiatric Research, New York University Medical Center, New York, New York, United States of America; Benito Menni Complejo Asistencial en Salud Mental, SPAIN

## Abstract

Inhibition of return (IOR) is an attentional mechanism that previously has been reported to be either intact or blunted in subjects with schizophrenia (SCZ). In the present study, we explored the neural mechanism of IOR in SCZ by comparing the target-locked N1 and P1 activity evoked by valid-cued trials with that evoked by invalid-cued trials. Twenty-seven schizophrenia patients and nineteen healthy controls participated in a task involving covert orienting of attention with two stimulus onset asynchronies (SOAs: 700 ms and 1200 ms) during which 64-channel EEG data were recorded. Behavioral reaction times (RTs) were longer in response to valid-cued trials than to invalid-cued ones, suggesting an intact IOR in SCZ. However, reduced N1 amplitude elicited by valid-cued trials suggested a stronger inhibition of attention from being oriented to a previously cued location, and therefore a relative inhibition of perceptual processing at that location in SCZ. These results indicate that altered N1 activity is associated with the preservation of IOR in SCZ and could be a sensitive marker to track the IOR effect.

## Introduction

Dysfunctional neurocognition is a core feature of schizophrenia (SCZ), which is associated with functional disability [1]. Some previous studies have found evidence for impairments in inhibitory control in schizophrenia [2–4]. The inability to inhibit responses to insignificant stimuli in patients with SCZ may lead to information overload, which may contribute to their symptoms, such as hypervigilance and difficulty in focusing attention [5]. In line with this, there is evidence that alterations in the hippocampal circuitry and frontal lobes are related to the failure of inhibitory attentional processes [5–10].

Of the many cognitive tasks that exist for studying inhibitory attentional deficits in schizophrenia, the task “inhibition of return (IOR)” is typically a good choice since research on IOR in healthy subjects has made the mechanism of IOR including the time course, spatial coding and functional significant much clearer [9,11]. IOR was first demonstrated by Posner and Cohen using a visual detection task [11,12]. A peripheral cue is followed by a target, which is presented in either the cued location or the un-cued location. When the interval between cue onset and target onset (stimulus onset asynchrony, SOA) is short, participants respond more quickly to the target at the cued location than at the un-cued location. In contrast, if the SOA is long (e.g., more than 300ms), participants respond more slowly to the valid-cued target than to the invalid-cued target [11,13]. The latter inhibitory aftereffect, named IOR, is thought to reflect an automatic, inhibitory mechanism that discourages attention from re-orienting back to the originally attended location [11,12,14–16].

However, research on IOR in schizophrenia patients has generated mixed and sometimes contradictory results [7,13,17–27]. Some researchers have reported that schizophrenia patients exhibited delayed or blunted IOR [19–24,27], while others have reported intact IOR in schizophrenia [13,17,18,25,26]. Our previous study found different time-course patterns of IOR between first-episode and chronic schizophrenia patients [7]. A recent meta-analysis indicated that conflicting findings of IOR in schizophrenia might be related to whether a single-cue or dual-cue procedure was used [9]. Delayed IOR in schizophrenia subjects was found in the single-cue procedure, while the time course of IOR in schizophrenia was more consistent with that of healthy controls in the cue-back procedure [9].

All the above findings concerning IOR in schizophrenia were inferred from behavioral reaction times (RTs), which are inherently ambiguous measures of inhibition and can be influenced by other processes [11]. The event-related potentials (ERPs) technique is beginning to serve as a complement to reaction-time measurement [28]. ERPs with an excellent temporal resolution of 1 ms can depict the time course of neural activity that is interposed between the stimulus and the response including both early sensory processes and late response-related processes [28]. ERPs have proven to be especially useful in investigating information processing in attentional paradigms [11,16,29,30]. Taylor and Klein have proposed two mutually exclusive “flavors” of IOR, the perception / attention flavor of IOR and motor theory of IOR, which have been widely investigated in healthy subjects [11,16,29,30]. The perception / attention flavor of IOR involves early sensory / perceptual processing in which attention is orientated towards invalid cued locations [29,30], and target-locked P1 and N1 are reduced in response to recently attended targets, suggesting the suppression of perceptual processing [29–31]. The motor theory of IOR connects IOR to response selection and motor processes [11,16,32], and target-locked lateralized readiness potential (LRP) has later onset on valid cued than on invalid cued trials, indicating an inhibition of premotor processing [29,30], or the oculomotor system [33,34].

No previous studies have examined the ERPs correlates of IOR effects in schizophrenia patients. In the present study, early visual ERP components (target-locked P1 and N1) were used to explore the neural correlates of IOR in SCZ. We hypothesized that the early visual ERP components would correlate with the cueing effect in IOR, and that P1 and N1 amplitudes would differ between trials (valid-cue trials versus invalid-cue trials) and between groups (schizophrenia patients versus healthy controls). Following Mushquash et al’s suggestion [9], the dual-cue procedure (the cue-back procedure) instead of the single-cue procedure was chosen in the IOR paradigm.

## Methods

### Participants and ethics statement

This study was conducted at the Shanghai Mental Health Center (SMHC). Twenty-seven inpatients with schizophrenia were enrolled in this study. A diagnosis of schizophrenia was confirmed by a research psychiatrist (D.L.) using MINI plus v 5.0 [35]. Subjects were either first-episode, drug-naïve schizophrenia patients or chronic schizophrenia patients who relapsed following drug withdrawal. All subjects had the capacity to provide informed consent, were in relatively stable clinical condition and appropriate for study participation determined by their treating psychiatrists. To rule out the short-term effects of antipsychotic medication on IOR, the clinical evaluations and ERP experiments were completed on the day of admission. Thus, when performing the experiments, no subject was treated with long-acting antipsychotic medications; all were antipsychotic free for at least 15 days before the experiment. Exclusion criteria for the study included: (1) inability to provide informed consent, (2) psychotic patients in unstable clinical conditions (e.g., being aggressive and uncooperative), (3) current substance abuse, (4) any other psychiatric diagnosis, (5) significant medical conditions including neurological disease, severe cardiovascular, hepatic, renal diseases, (6) pregnancy or breastfeeding.

Nineteen healthy controls (HC) were recruited from the local community. All of them completed the structured clinical interview by a research psychiatrist using MINI plus v 5.0. Those with any psychiatric disease, neurological disease, or a positive family history of psychiatric disease were excluded. Written informed consent was obtained from each participant. If the participants had no capacity to consent, written informed consent was obtained from their legally authorized representative on the behalf of participants. The study protocol was approved by the SMHC Ethics Committee in compliance with the Helsinki Declaration.

Clinical symptoms were assessed using the Positive and Negative Symptom Scale (PANSS) [36–38]. The severity of illness was assessed by the Clinical Global Impressions-severity scale (CGI) [39].

### Materials and procedure

**Fig 1:**
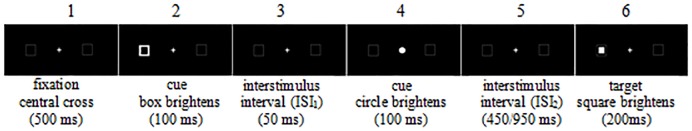
The procedure of modified IOR paradigm. Subjects were instructed to focus on the central cross and maintain fixation throughout the first five frames of the trial. Frame 1: the start of each trial, fixation on the central cross; Frame 2: a peripheral cue was presented randomly to the left or right of fixation; Frame 3: the cue offset for a brief inter-stimulus interval (ISI); Frame 4: then central fixation cue (the cue-back procedure); Frame 5: variable ISI_2_ including 450 ms and 950 ms; Frame 6: the target appeared in the cued or uncued location with equal probability. The entire experiment consisted of 320 trials.

For a previous study [7] we developed a modified IOR paradigm based on the methods of Posner and Cohen [12]. The dual-cue task is illustrated in Fig 1. All stimuli were presented in white on a black background. Each trial began with a centrally presented cross lasting 500 ms. Subjects were instructed to focus on the cross. A peripheral cue was presented for 100 ms randomly to the left or right of fixation with equal probability. Then the central cue was presented by brightening the central cross for 100 ms. The aim of this was to re-orient the participants’ attention to the central fixation. The interstimulus interval (ISI_1_) between the peripheral cue offset and the central cue onset was 50 ms. The interstimulus interval (ISI_2_) between the central cue and the target onset randomly varied between 450 ms and 950 ms, corresponding to 2 different levels of Stimulus-onset asynchrony (SOA). The target stimulus was a white square presented in the cued or uncued location with equal probability. The subjects were asked to press a button labeled “1” as soon as the target appeared. The target remained on for 200 ms and was followed by a 1300 ms black display during which subjects responded.

The experiment was performed in a sound-attenuating, electrically shielded chamber with dim illumination. Participants were seated about 1-m from the screen. They were instructed to sit quietly and focus on the center of the monitor. The subjects were asked to press the “1” button as soon and correctly as possible when the target appeared. The stimuli were presented with a rest period of 1 min between blocks.

### EEG recording and data preprocessing

The electroencephalogram (EEG) was recorded from 64-channel surface electrodes mounted in an elastic cap (BrainCap, Brain Products Inc., Bavaria, Germany) including two pairs of vertical and horizontal electro-oculograhy (EOG) electrodes, which were simultaneously recorded to monitor eyes movements and blinks. Scalp impedance for each electrode was kept below 5 kΩ. Data recording was sampled at 1000 Hz with a reference to the tip of the nose.

Artifacts from vertical and horizontal eyes movements and blinks were removed offline by an ocular correction algorithm using Brain Vision Analyzer (Brain Products Inc., Bavaria, Germany) [40]. Artifact-free data were filtered with a zero phase-shift IIR band-pass filter of 0.01–40 Hz (24 dB/Oct). EEG was segmented separately from 100 ms before the stimulus onset to 800 ms post-stimulus, and then baseline corrected to the first 100 ms of each epoch. Segmentations with errors and those RTs outside of 200 to 1500 ms were excluded from the analysis.

Individual EEG segmentations were averaged separately for each category of stimuli at electrodes PO7, PO8 and Pz. Since the activity at the contralateral occipital electrode is more active when the stimulus is displayed in the left/right visual field, we only included the contralateral occipital N1 and P1 activity for analysis. The difference waveforms were obtained by subtracting the grand average waveforms evoked by invalid-cued trials from those evoked by valid-cued trials.

Grand averages were smoothed with a 0.5–30 Hz band-pass filter. Individual peak amplitudes of P1 and N1 components were obtained according to the local maximum within the P1 time-window (100–170 ms) and the N1 time-window (160–220 ms), respectively. Individual peak latencies of P1 and N1 were the times of the P1 and N1 peaks. These values were obtained for each category of stimuli and the difference waveforms.

### Statistical analysis

Trials with too fast of a response (RTs＜200 ms) or too slow of a response (RTs＞1500 ms) were excluded. Trials with error responses were also excluded from further analyses. The variables including age, education, behavioral RTs, and N1 amplitudes and latencies were normally distributed by Kolmogorov-Smirnov test.

Comparisons of demographic variables between groups were performed using independent *t* tests for continuous variables and chi-square tests for categorical variables.

The main aim of the present study was to examine whether the IOR effect differed between SCZ patients and healthy controls. Thus, behavioral RTs, amplitudes and latencies of P1 and N1 at the contralateral occipital electrode were analyzed using repeated-measure ANOVA for two within-group factors Cuing (valid-cued vs. invalid-cued) and SOA (700 ms vs. 1200 ms), and between-group factor Group (schizophrenia group versus healthy control group). In this statistical design, the main effects of Cuing, SOA and Group, and the interactions of Cuing×Group, Cuing×SOA×Group were examined. The main effect of Cuing reflected the IOR effect described by different performances between in valid-cued trials and in invalid-cued trials. The interaction of Cuing×Group reflected how the IOR effect (the Cuing effect) differed between groups.

When there was a significant interaction of Cuing×Group, subsequent simple main effects test of Cuing was performed by comparing RTs, amplitudes and latencies of P1 and N1 of the valid trials with those of the invalid trials within each group at each SOA. Subsequent main effects test of Group was also performed by comparing the group differences in valid-cued and invalid-cued trials at each SOA, respectively. Bonferroni adjustments were used for multiple comparisons.

## Results

### Demographic and clinical characteristics

Table 1 displays demographic and clinical characteristics of schizophrenia patients and healthy controls. There were no significant differences between the 2 groups in gender, education and age. For the schizophrenia group, the PANSS total score and CGI-SI score were 86.1±10.9 and 5.7±0.7, respectively; the illness duration was 9.4±7.8 years; and the number of episodes was 2.7±0.9.

**Table 1:** Demographic and clinical characteristics of schizophrenia patients and healthy controls (Mean±S.D.).

Characteristics	Schizophrenia patients	Healthy controls	Statistical significance
Cases	27	19	N.A.
Handedness (left/right)	0/27	0/19	N.A.
Age (years)	34.2±9.6	30.6±7.2	t(1,44) = -1.37, P = 0.18
Education (years)	12.8±2.8	14.0±2.7	t(1,44) = 1.45, P = 0.15
Illness duration (years)	9.4±7.8		
Number of episodes	2.7±0.9		
PANSS-total	86.1±10.9		
PANSS-positive	22.2±5.1		
PANSS-negative	22.1±6.2		
PANSS-general	41.9±6.3		
CGI-severity	5.7±0.7		
Gender			
Male	19	10	χ^2^ = 1.51, P = 0.22
Female	8	9	

PANSS-total: total scores of Positive and Negative Syndrome Scale. PANSS-positive: scores of positive symptoms subscale of Positive and Negative Syndrome Scale. PANSS-negative: scores of negative symptoms subscale of Positive and Negative Syndrome Scale. PANSS-general: scores of general psychopathology subscale of Positive and Negative Syndrome Scale. CGI-severity: Clinical Global Impression.

### Behavioral results

**Fig 2:**
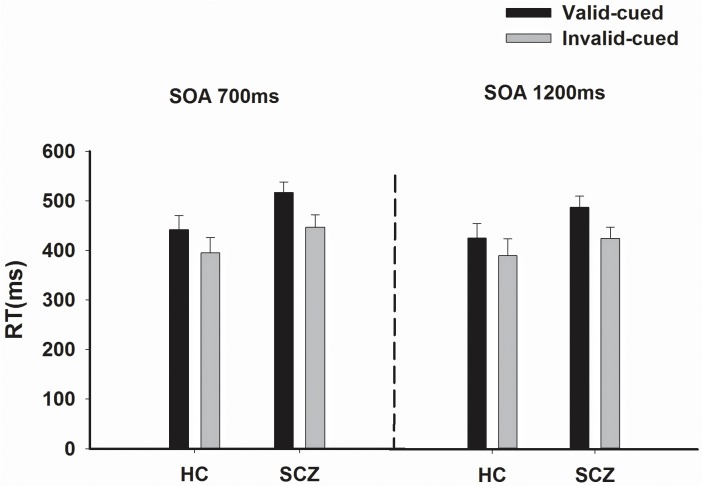
RTs in the covert orienting of attention task with exogenous cues in the schizophrenia group (SCZ) and the healthy control group (HC). Both the main effects of Cuing and SOA for RTs were significant (p<0.01).

Mean correct RTs stratified by groups, SOA and cuing are presented in Fig 2. A significant SOA main effect was observed [F(1,44) = 27.11, *p* < 0.001], RT at SOA 700 ms was significantly longer than the RT at SOA 1200 ms. As expected, the cue main effect was significant [F(1,44) = 79.30, *p*<0.001], RTs were much shorter in the invalid-cued trials (414.20±19.52 ms) as compared to those in the valid-cued trials (467.99±17.64 ms). Although the group main effect was not significant [F(1,44) = 2.34, *p* = 0.13], the interaction of cuing and group was significant [F(1,44) = 4.41, *p*<0.05].

In the valid-cued trials, subsequent simple main effect of Group with Bonferroni adjustment revealed that schizophrenia patients had longer RTs compared with healthy controls at SOA 700 ms (*p*<0.05), and tended to have longer RTs at SOA 1200 ms (*p* = 0.09). There were no significant group differences for RTs in the invalid-cued trials at both SOAs (*p* = 0.20 and *p* = 0.37, respectively). These results showed that RTs differed between two groups in the valid-cued trials but not in the invalid-cued trials.

Subsequent simple main effect of Cuing with Bonferroni adjustment were also performed to determine the cuing effects at each SOA within each group by comparing RTs between the valid-cued trials and invalid-cued trials. The results are presented in Table 2. In both schizophrenia patients and healthy controls, the RTs in valid trials across all SOAs (700 ms and 1200 ms) were longer than in invalid trials, which indicated that schizophrenia patients had a normal pattern of IOR for SOA 700 ms and 1200 ms.

**Table 2:** The cuing effect and inhibition of return (IOR) at each SOA in schizophrenia patients and healthy controls (Mean±S.D.).

SOAs(ms)	700ms	1200ms
Schizophrenia patients		
Valid-cued trials	516.7±110.6	488.1±114.4
Invalid-cued trials	446.5±129.7	425.3±116.7
Cuing effect	-70.1	-62.8
*p*	*p* < 0.0001	*p* < 0.0001
Healthy controls		
Valid-cued trials	441.5±124.8	425.7±129.2
Invalid-cued trials	394.7±134.7	390.3±148.1
Cuing effect	-46.8	-35.4
*p*	*p* < 0.0001	*p* < 0.0001

SOA: stimulus onset asynchrony = time from onset of cue to onset of target. Cuing effect: RT_invalid trials_-RT_valid trials_, which indicated facilitatory effect or inhibitory effect of cuing, and the facilitatory cuing effects on RT was indicated as positive, inhibitory cuing effect on RT was indicated as negative.

### ERP results

**Fig 3:**
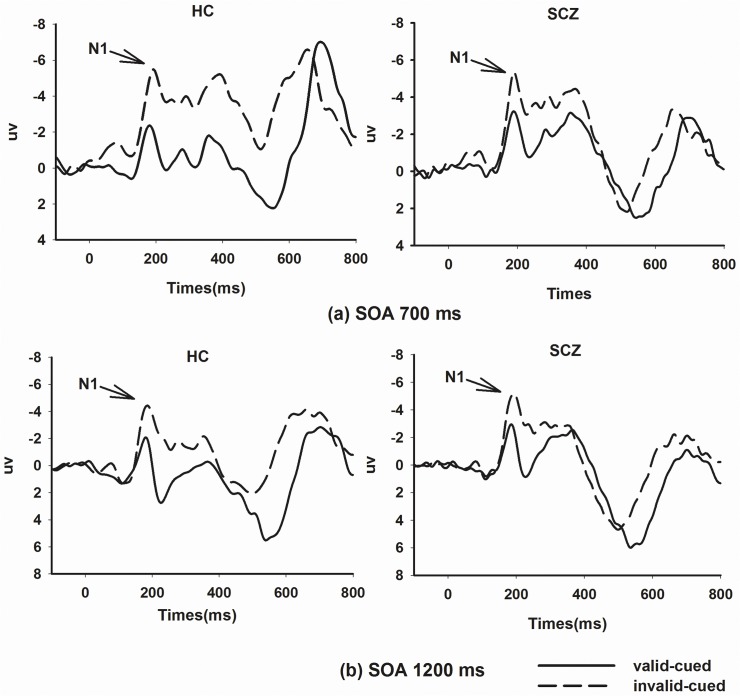
The grand ERPs at contralateral electrodes. (a) at SOA 700 ms: left, enhanced N1 component in invalid-cued trials than in valid-cued trials in the healthy control group (HC); right, significantly enhanced N1 component in invalid-cued trials than in valid-cued trials in the schizophrenia group (SCZ); (b) at SOA 1200 ms: left, no significant cuing effect for N1 component in the HC group; right, significantly enhanced N1 component in invalid-cued trials than in valid-cued trials in the SCZ group.

P1 and N1 components evoked by the target were analyzed. A significant main effect of Cuing was observed only on the N1 component as shown in Fig 3. There were no significant main effects of Cuing or Group on the P1 component so that P1 results are not shown.

### N1 amplitude

A main effect of Cuing was significant on the N1 amplitude [F(1,44) = 16.49, *p*<0.001]. The N1 amplitude evoked by the valid-cued trials (-4.67±0.35μv) was reduced compared to that evoked by the invalid-cued trials (-6.18±0.46μv). There were no significant main effects of Group [F(1,44) = 0.14, *p* = 0.71] or SOA [F(1,44) = 1.55, *p* = 0.22]. The interaction of Cuing and Group was significant [F(1, 44) = 4.29, *p*<0.05].

Subsequent simple main effect of Cuing with Bonferroni adjustment was performed to determine the cuing effects at each SOA within each group. The results are presented in Table 3. In schizophrenia patients, N1 amplitude evoked by valid-cued trials was lower than that evoked by invalid-cued trials across both SOA 700 ms (*p*<0.001) and 1200 ms (*p*<0.01). In healthy controls, no significant cuing effects on N1 amplitudes were observed at both SOA 700 ms (*p* = 0.20) and 1200ms (*p* = 0.47). The results reflected that the interaction presented different cuing effects on N1 amplitude between two groups.

**Table 3:** The cuing effects on N1 amplitude (μv) at each SOA in patients with schizophrenia and healthy controls (Mean±S.E.).

SOAs(ms)	700ms	1200ms
Schizophrenia patients		
Valid-cued trials	-4.12±0.60	-4.18±0.57
Invalid-cued trials	-6.45±0.68	-6.42±0.78
*p*	*p* < 0.001	*p* < 0.01
Healthy controls		
Valid-cued trials	-5.56±0.67	-4.82±0.42
Invalid-cued trials	-6.50±0.64	-5.36±0.70
*p*	*p* = 0.20	*p* = 0.47

SOA: stimulus onset asynchrony = time from onset of cue to onset of target.

### N1 latency

The cuing effect on the N1 latency was significant [F(1,44) = 6.62, *p*<0.05]. The N1 latency evoked by valid-cued trials (189.94±1.72 ms) occurred much earlier than that evoked by invalid-cued trials (194.08±1.71 ms). There were no significant main effects of SOA [F(1,44) = 0.02, *p* = 0.90] or Group [F(1.44) = 1.12, *p* = 0.30] on the N1 latency.

## Discussion

Dysfunctional neurocognition in SCZ often predicts long-term functional disease outcome [3,41]. Structural and functional alterations related to attention deficit in patients with SCZ have been observed in the dorsolateral prefrontal cortex, the insula, the anterior cingulate gyrus, hippocampus and ventral striatum [9,41]. Attention is an area of major impairments in SCZ [42,43]. In the present study, IOR was intact at both SOAs of 700 ms and 1200 ms in schizophrenia patients. Inhibited orientating to previously cued locations compared to novel locations was indexed by facilitated RTs in both schizophrenia patients and healthy controls. However, there was a significant interaction of Cuing and Group on N1 amplitude. N1 amplitude evoked by invalid-cued trials was significantly enhanced compared to that by valid-cued ones in SCZ. This indicated that more resources were involved in perceptual processing to preserve the function of IOR in SCZ.

Previous behavioral studies in IOR have examined attention deficit in SCZ with mixed findings [7,9,13,21,23,24,27,29,44,45]. The IOR effects in SCZ vary with the cue-target task procedure (single cue task or cue back task), illness duration and severity, and medications [7,9,46]. Our present study found RTs in response to the invalid-cued trials were much shorter than those in response to the valid-cued trials on both SOAs (700 ms and 1200 ms), suggesting normal levels of IOR in SCZ. In addition, RTs were facilitated as SOA increased from 700 ms to 1200 ms in both groups. Our findings are consistent with Sapir et al.’s report that IOR was normal in SCZ using the cue back paradigm [27,47]. Further, our study showed a significant interaction between groups and cuing conditions on RTs. SCZ patients had longer RTs compared with healthy controls in the valid-cued trials, but there were no significant between-group differences of RTs in the invalid-cued trials. Our findings suggested that SCZ subjects may not have difficulty in disengaging attention from a previously directed location but they may have difficulty in attention maintenance [48].

Although IOR in SCZ appeared intact as measured by RTs, different N1 activities were found in SCZ. Enhanced N1 amplitude was elicited by invalid-cued trials compared to that by valid-cued ones of both SOAs in SCZ. Studies of IOR in healthy controls suggested that IOR might be associated with perception and attention [16,29]. Enhanced N1 amplitude by invalid-cued trials as compared to valid-cued trials suggested a mechanism that inhibited attention from being oriented to a previously cued location would result in a relative inhibition of perceptual processing at that location [29,30]. The hyperactivity of N1 activity was accompanied by RT facilitation [29]. We also found a significant interaction of Group and Cuing on N1 amplitude, which represented different cuing effects between SCZ patients and healthy controls. The differences in N1 activities between valid-cued trials and invalid-cued trials increased in SCZ, suggesting more resources were involved in the perception processing [29,30]. Thus, IOR in SCZ might be preserved at the cost of allocating more resources.

Our results suggested that N1 amplitudes were more sensitive than RTs. In the future, we will continue to study different cuing effects of N1 activity between first-episode and chronic SCZ, and between SCZ with and without antipsychotic medicine treatment. This line of studies will help confirm the utility of using N1 amplitude as an endophenotype for attention deficit in SCZ.

In addition to a small study sample size, another limitation of the present study is that SOAs shorter than 700 ms were not studied and cuing effects of SOA 700 ms differed between our present and previous studies [11,49]. Studies on the time course of IOR found delayed onset in SCZ (SOA of 700–800 ms versus 300 ms in healthy controls) [19,23]. Whether there was an intact IOR with SOA of 700 ms is uncertain, but this may explain the difference in our current and previous findings.

## Conclusion

In conclusion, the present study is the first to report ERP consequences of IOR in SCZ suggesting the neural correlates of the IOR effect in SCZ. The behavioral RTs showed an intact IOR effect in schizophrenia patients, whereas the ERP demonstrated enhanced target-locked N1 amplitude elicited by invalid-cued trials in SCZ. The differences in N1 activities between valid-cued trials and invalid-cued trials increased in SCZ, suggesting more resources were involved in the perception processing. Our results suggest that N1 activity is associated with the preservation of IOR in SCZ and could be a sensitive marker to track the IOR effect.
